# Kinematic Analysis of Canoe Stroke and its Changes During Different Types of Paddling Pace – Case Study

**DOI:** 10.2478/v10078-011-0036-7

**Published:** 2011-10-04

**Authors:** František Zahálka, Tomáš Malý, Lucie Malá, Martin Doktor, Jan Větrovský

**Affiliations:** 1 Charles University, Faculty of Physical Education and Sports. Prague. Czech Republic

**Keywords:** Canoe, kinematic analysis, paddling technique, 3D analysis

## Abstract

The aim of the study was to describe and evaluate movements of an elite canoeist when different paddling paces are applied. One of the tasks consisted of finding differences in time-space characteristics of selected markers in the referencing system canoeist’s body - canoe. 3D kinematic analysis was used for identification and comparison of selected parameters. The study confirmed that an elite canoeist has a high level of movement similarity in all types of stroke rates; differences were mainly found in time sequence of applied strokes. To evaluate properly the racing stroke rate, start, flying start, 200m pace, 500m pace, and 1000m pace were chosen. One of the evaluated parameters was the boat velocity that was 2.1–4.5 ms^−1^ at start, 3.2–5.2 ms^−1^ at flying start, 3.9–6.1 ms^−1^ at 200m pace, 3.9–5.9 ms^−1^ at 500m pace and 3.0–5.4 ms^−1^ at 1000m pace. Vertical change of the position of the right hand was 0.77 m at start, 0.73 m at flying start, 0.87 at 200m pace, 0.89 at 500m pace and 0.81 m at 1000m pace.

## Introduction

The aim of the study was to describe and evaluate an elite canoeist movement when different paddling paces are applied and to find out how movement stereotype changes as pace changes. Being technically well prepared is important for motion of the canoe in the water and for sport performance during the race. It mainly involves technically correct strokes in terms of effectiveness. The main moving mechanism is a paddle stroke and it is also very important to technically master the transfer of maximal force to the paddle well (Baker et al., 1999). Technically correct stroke is a keystone on which we may build the athlete’s performance in sprint canoeing (Doktor, 2001). The stroke can be divided into several successive parts such as: preparation phase, catch phase, power phase, recovery phase and manoeuvring the canoe. Stroke begins in a basic position when the athlete is kneeling while the second leg is flexed at the knee with angle ranging from 80° – 120 ° degrees. Relaxation is characteristic for the phase after the paddle has been pulled out of the water. When maintaining a slower pace, the relaxation phase is more distinct, getting shorter with higher frequency should be, nevertheless, still be present (Sperlich & Baker, 2002). The canoeist’s movement (upper limbs and trunk) continues from the basic position in concordant direction with canoe movement. The shoulder of the upper extremity, placed on the paddle nearer the blade, is moving forward. The shoulder of the upper extremity holding the paddle grip creates conditions for paddle entry in the water at the right angle. The lower extremities movements together with hips make the fluent and uninterrupted transition to the phase of paddle entry. Fluency of the movement is important for the continuous forward movement of the canoe (Sanders, 1998). The paddle movement following the catch phase continues in forward movement to the power phase, which is the most important moving mechanism of the whole stroke. The canoeist should try to reach the highest force transfer through the paddle to the boat. The whole stroke finishes with pulling the paddle out of the water, trying not to change the direction of the forward canoe movement but to enhance the forward canoe movement by letting the boat glide. Canoe conducting is performed at the final part of the power phase (Baker et al., 1999; Doktor, 2001). Besides basic phases, shared in the overall movement, it is possible to detect differences in paddling techniques. In sprint canoeing - C1, we may differentiate 4 basic paddling techniques: paddling with dynamic vertical body motion and rotation, paddling with trunk rotation, technique with fixed hip and technique with hip rotation (Szanto, 1994).

## Material and Methods

The participant was an elite athlete in the active racing period, a medallist at world championships and Olympic Games, which determines mastering the technique at the top level. 3D kinematic analysis was used for the athlete’s monitoring (Janura & Zahálka, 2004) during two days in the racing period. We used 5 video cameras using Mini-DV format. TEMA Bio 2.3 software was used for data assessment. Recording frequency was 50 half shots per second. The participant was monitored during training sessions at start, flying start, distance of 200 m, 500m and 1000m and 2 × 10m. For kinematic analysis, we used 6 attempts at distances of 500 m and 1000 m and 8 attempts at 200 m, start and flying start. Totally 12, attempt of 16 images were taken. The measured area was calibrated by cuboid with dimensions 1 × 2 × 4 m. Calibration was chosen as 12-point and traceability reconstruction of spatial coordinates was carried out by implementing spatial coordinates into equations with the calculated coefficients of DLT for obtaining deviations from the real location of points and calculated values and for determination of measurement error. Real projection room had a width of 10 m, which for average deviation 0.0296, calculated from all deviations, means 0.3 % width of the scanned image.

**Figure 1:**
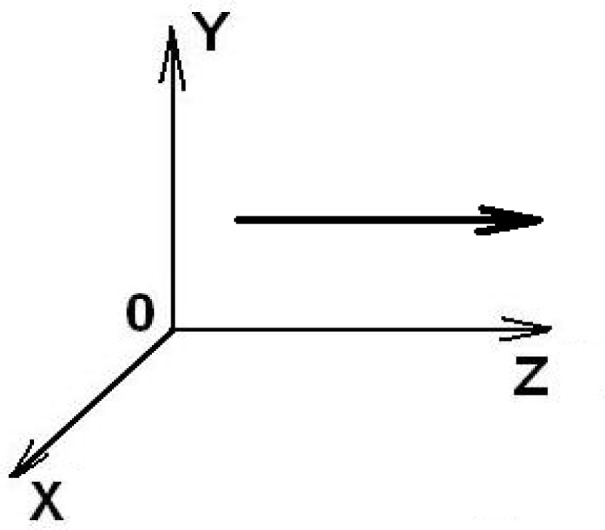
Established coordinate system showing the movement in a positive direction

**Figure 2:**
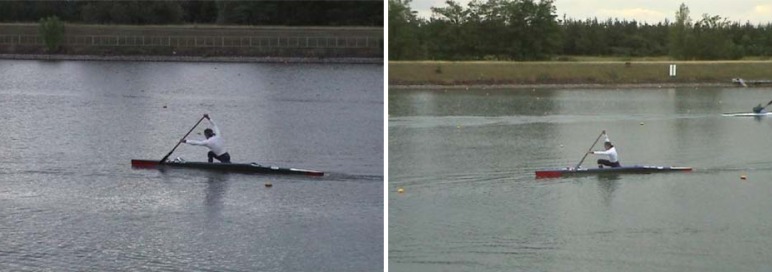
The view of the participant when passing 10 m distance from both sides

The image (Figure 1) presents an established coordinate system for spatial analysis, where the arrow indicates the direction of movement. When interpreting values, it is essential to realize that the movement is shown according to the established coordinate system, therefore, from left to right, although in the examples of real images the athlete goes from right to left in the racetrack (Figure 2).

For the analysis, major points on the body of athlete and the boat were selected. These points were: right shoulder, right hip, right knee, right elbow, right hand, left shoulder, left elbow, left hand, head, tip of the boat, the stern of the boat and the segment of „right tibia“. This was designed as a line between the point representing the right knee and the middle of the segment of the lower limb just above the upper edge of the boat (ankle is not visible in the boat). Selected points named as right and left hand were placed so that they represent the centre point where holding the paddle. These points were selected for the characteristic of position of the upper limbs with the possibility to assess the position of the paddle. Point called the head represents the centre of the participant’s head. All points were entered subjectively by a trained operator and the error does not exceed the reconstruction error of the scanned area (Janura & Zahálka, 2004).

## Results

### Kinematic analysis of a stroke – identification of important phases

**Figure 3:**
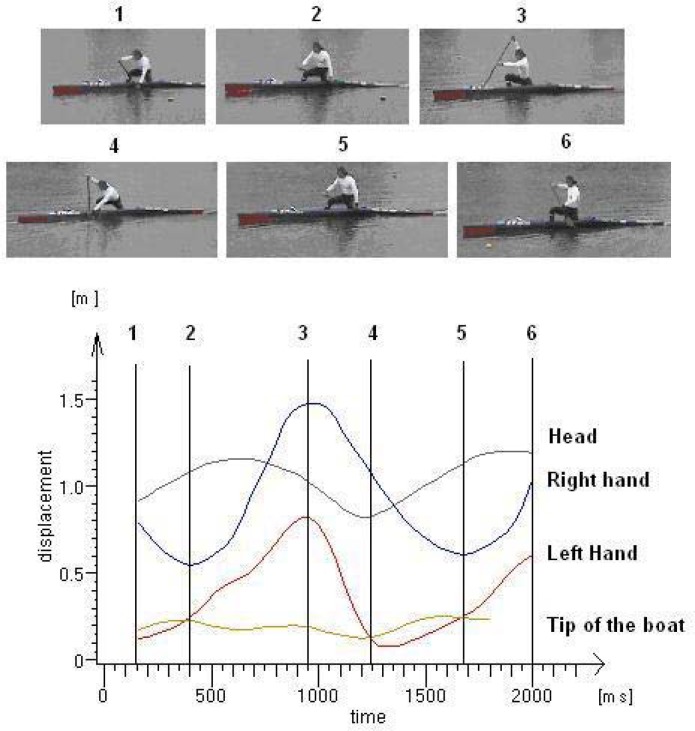
Trajectories of selected points on Y axis – vertical change of position

The image (Figure 3) presents the displacement of selected points in the Y axis (vertical change) versus time. On the graph, important moments related to particular phases of the stroke are marked. Moment 1 represents finishing of the previous stroke; moment 2 refers to the end of the stroke and the moment when the right hand is in the lowest vertical position and when the air phase begins until the paddle enters the water (the first contact of the paddle and water) which is indicated as moment 3. There are the points representing both hands in the maximum vertical position. Moment 4 represents a situation where the paddle is inserted in the water, the position of the paddle to the water is almost in an upright position and the most effective part of the stroke begins. Moment 5 represents the final phase of the stroke, as well as the previous moment 2. In moment 6, the athlete transfers the paddle to the starting position entering the paddle in the water.

### Kinematic analysis of starts

The start is a very important part of the race and hence, the paddling technique is very substantial. At the start, the boat is at zero velocity and therefore, it is necessary to put the boat quickly into motion. When using flying start, the pace is similar to solid start, but the distance between particular strokes is greater due to higher initial velocity. In addition to the values representing the tip of the boat, all other segments have almost constant values (Table 2). It is thus obvious that at this pace, when the participant must give the boat as much speed as quickly as possible, his technique is, in all the strokes, almost identical. Vertical deviation between the lowest and highest position of the right hand at the start is 0.77m. In the flying start this value was about 0.04 m lower. The left hand moved in the vertical plane during one stroke about 0.57 m (start), i.e. 0.55 m (flying start). The head moved by 0.26 m at the start and 0.25 at the flying start. The tip of the boat during the stroke moved in the vertical direction by 0.13 m (start), i.e. 0.10 m (flying start). The biggest difference was seen at the distance the boat ran between strokes. In the case of start, this difference made up 2.55 m. For the flying start it was 4.14 m, which means a difference of 38.4%. Deflection of the tip of the boat at the beginning and end of the monitored distance (10 m) was in both types of takeoffs equal (0.60 m). Trajectory is without great fluctuation and shows high similarity. In the right hand we noticed an expressive movement caused by pulling the paddle out of the water and the subsequent transfer of the paddle forward. This lateral displacement was 0.7 m at the start and 0.8 m at the flying start. At the starting pace, boat velocity increases with each stroke and during the monitored distance its maximal velocity increased from 3.6 ms^−1^ to 4.5 ms^−1^. Gradually minimum velocities increase as well from 2.1 ms^−1^ up to 2.9 ms^−1^ (Table 1). This trend was also registered at the flying start when boat velocity increased from 3.2 ms^−1^, i.e. 3.4 ms^−1^ at the minimum values to 4.9 ms^−1^ i.e. 5.2 ms^−1^ at the maximum values. An interesting moment of velocity is the interval beginning around the time 850 ms (start), i.e. 800 ms (flying start) and regularly repeating in the same trend. From this moment there is an increase in boat velocity and velocity of the point representing the athlete’s head nearly reaches the maximum value. The left hand accelerates after the power phase and reaches the maximum value before the paddle enters the water. At the moment when the boat has the lowest velocity, velocity of the head and torso is before its peak. Transfer of the upper extremity and torso in the frontal direction affects the movement of the boat so that its velocity will increase. At the moment when velocities of the head and both upper extremities begin to fall, boat velocity starts to increase. Deceleration rate of the head and both upper extremities is caused by the fact that the paddle is already in the water and the power phase is being performed. The boat is at this stage powered by paddles and in consequence of an increased resistance to the paddle, velocities of the head and both upper extremities are falling. The boat gets maximum velocity when velocities of the head and the left upper extremity decrease to the minimum level. Since at this point the paddle is in the optimal position for the power phase – the entire blade in the water and vertically to the surface, velocity is highest. At the end of the power phase boat velocity begins to fall and reaches its minimum at the end of the air work phase.

### Kinematic analysis of various paces (200 m, 500 m, 1000 m)

**Figure 4:**
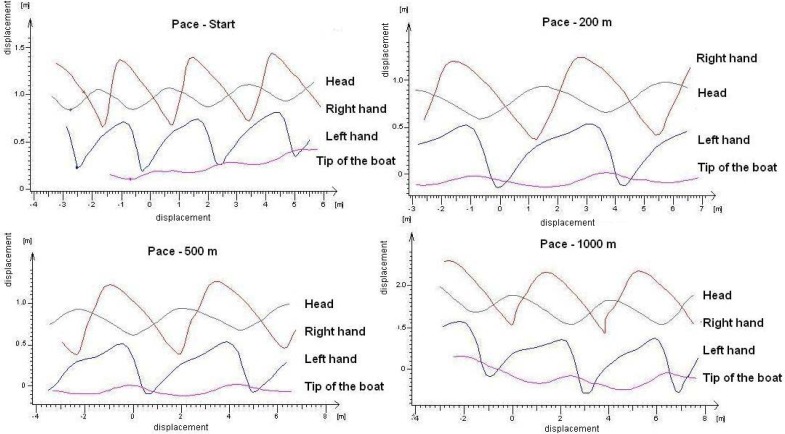
Trajectories of selected points in plane ZY at different types of paddling pace

In all paces, it is important to give the boat maximum velocity and maintain it throughout the whole race. The measured values indicate persistence of technique implementation. In case of distances of 200 and 500 m, segment trajectories are very similar in all strokes. In case of 1000 m, where the recording was obtained at 800 m after the start, the slight decrease in velocity is apparent, which is likely caused by fatigue. When evaluating specific changes it can be noticed that there is a slight decrease of values in the monitored segments. Vertical differences in segments have, however, a steady downward trend. Measured values of differences are almost constant. It is therefore evident that even at this pace, when the participant has to overcome considerable fatigue, his technique is consistent. When comparing vertical deviations between the lowest and highest position of the right hand we found at the shortest distance (200 m) a value of 0.87 m. In case of medium distance it was 0.89 and at the longest, distance it was 0.81 m. The left hand moved in a vertical plane within one stroke of 0.69 m (200 m), 0.63 m (500 m) i.e. 0.70 m (1000 m). Minimum displacements were observed in the movement of the head in the vertical direction (0.30 at 200 m pace, 0.33 at 500 m pace and 0.32 at 1000 m pace). Almost the same values were found in the changes of the tip of the boat position during the stroke in the vertical line at distances of 200 and 500 m (0.17 m, i.e. 0.14 m). Also in these paces, we noticed comparable distance the boat runs between individual strokes (4.37 m, i.e. 4.30 m). At the distance of 1000 m, the tip of the boat oscillated in the range of 0.10 m – 0.22 m. The distance the boat covered between individual strokes is 3.93 m which is by 10.07 % shorter when compared to 200 m pace, i.e. by 8.61 % less than at 500 m pace. Deflection of the tip of the boat in the transverse direction at the beginning and end of the monitored distance (10 m) was 0.50 m at the shortest distance. At 500 m pace during the monitored distance (10 m) the boat executed two displacements, which could have been caused by the wave. Trajectory displacement was 0.30 m. The tip of the boat at the distance of 1000 m carries a greater curve to the right during the strokes than at the shorter distances, which is due to lower activity in the boat manoeuvring. Without active manoeuvring, deviation from the direct path of the boat may reach 2.40 m. This value may seem relatively large, but because the participant has rich experience with the racing pace, deflection of the boat is probably more preferable to him than decreasing boat velocity by manoeuvring. At all paces, we may notice significant movement in the right hand caused by pulling the paddle out of the water and subsequent transfer of the paddle in the forward direction. This lateral deviation was 0.80 m (200 m pace and 1000 m pace) and 0.85 m (500 m pace). At 200 m pace, the maximum velocity 6.1 ms^−1^ is maintained at the same level throughout the whole distance. The minimum velocity stabilised at 3.9 ms^−1^ (Table 1). At 500 m pace, the maximum velocity 5.9 ms^−1^ is maintained at that level throughout the whole distance. The minimum velocity stabilised at 3.9 ms^−1^. During the monitored distance at 1000 m pace, the maximum velocity decreased from 5.4 ms^−1^ to 5.0 ms^−1^. Gradually also the minimum velocity decreased from 3.5 ms^−1^ up to 3.0 ms^−1^. In times of about 900 ms (200 m pace, 500 m pace) i.e. 700 ms (1000 m pace) the beginning of a trend that is regularly repeated is apparent. From that moment on, boat velocity rises and velocity of the point representing the athlete’s head nearly reaches the maximum value. In the left hand there is a trend of accelerating after the power phase and the maximum values are reached before entering the paddle into the water. At a time when boat velocity is lowest, velocity of the head and torso is before its peak. Transfer of the upper extremities and torso in the frontal direction affects the movement of the boat so that its velocity will increase. At a moment when velocities of the head and both upper extremities begin to fall, boat velocity begins to rise. Deceleration of the head and both upper extremities is caused by the fact that the paddle is already in the water and the power phase is being performed. The boat, at this stage, is powered by paddles and due to an increased resistance to the paddle the velocity of the head and both upper extremities are falling. The boat reaches its maximum velocity at the moment when the velocity of the head and the left upper extremity reaches its minimum level. Since at this point the paddle is in the optimal position for the power phase – the whole blade in the water and vertically to the surface – its velocity is highest. At the end of the power phase, the boat velocity begins to decrease and reaches its minimum at the end of the air work phase. Trajectories of the selected points in plane ZY at different types of paddling pace and number of strokes are reported in Figure 4. In the presented graphs, we can notice that, at start and flying start, the athlete performs 3 strokes at the distance of 7.5 m which means 2.5 m per one stroke. At all other paces, the athlete performs 2 strokes at 9 m, which makes 4.5 m per stroke. Figure 4 demonstrates graphical similarity of strokes at different paces; specific data of vertical changes in the selected parameters are presented in Table 2.

## Discussion

Measured values confirmed that the boat increases its velocity during the power phase and also during this phase reaches its maximum velocity. Finally the boat slightly slows down as a result of correction of boat direction. The fact that the boat is already picking up the velocity at the end of the air work phase is caused by the movement of the torso in an individual implementation of the movement pattern. During the air work phase, the participant is preparing for another stroke. At this phase, the paddle is in the air. The participant has to transfer the paddle for another stroke as quickly as possible. The main propulsion of the boat is the stroke and since at this stage the paddle is not in the water the boat loses its velocity (Sanders & Kendal, 1992).

At all paddling paces the trend towards slowing down is identical. In the final part of the power phase, in manoeuvring, the boat slightly loses its velocity. Decelerating trend is apparent during the whole air work phase and the boat reaches its minimum velocity at this stage. But at the end of this air work phase, velocity begins to increase thanks to the movement of the torso (Aitken & Neal, 1992). Divergence of technical performance of strokes at different types of paddling paces has been proved, but individual deviations in the parameters are only slight. The time sequence of individual strokes differs and depends on the chosen pace. Paddling technique at different paces does not show significant changes and therefore is not affected by location or movement of the boat. Change of the movement of the canoe is more significant only at starting pace, which is due to a higher frequency of individual strokes in order to get the highest possible velocity in the shortest possible time. The results indicate a similar trend of the movement of the boat in the XY plane and XZ plane. The canoe velocity during the air work phase and preparation for the next stroke decreases, but thanks to the forward movement of the torso at the moment of preparation for entering the paddle into the water the boat begins to pick up velocity before the moment when the paddle enters water. The boat velocity starts to increase before the paddle is inserted into the water, which is probably caused by the forward movement of the torso and transfer of weight to the lower extremities. Here is the biggest difference compared to previous researches (Plagenhoef, 1979; Baker et al., 1999), where during the air work phase only slowing down occurs. At the time of entering of the paddle into the water the canoe is already picking up velocity and during the power phase it is still accelerating. The canoe reaches its maximum velocity at the moment when the paddle is in the optimal position (vertically to the surface) (Janura et al., 2000). Possibly due to the movement of the torso, which straightens out and acts against the direction of the boat and also due to fact that the stroke is effective no more, the canoe loses its velocity. The velocity of consecutive strokes is higher at the start than at other paddling paces. This is caused by the need to obtain the highest possible velocity in the shortest possible time. At the starting pace, individual strokes follows within 700 ms. At 200 and 500 m paddling paces, this time is about 900 ms. At 1000 m pace, it is 1100 ms. Range of motion, in other words differences between the highest and lowest positions of individual segments, varies only slightly at different paces. These differences are from 0.77 m to 0.95 in the right hand at 200, 500 and 1000 m. At starting paddling paces this range is 0.73 – 0.77 m. The left hand moves by 0.55 – 0.70 m in the vertical direction. The head moves by 0.25 – 0.33 m. The movement of the tip of the boat is 0.14 – 0.22 m at distance paces; at the start these differences are only 0.10 – 0.13 m, which is due to the rapid sequence of individual strokes and smaller vertical movement of the canoeist. The movement of the boat, i.e. its velocity, is influenced mostly by the power phase. The canoeist powers the boat in the forward direction by means of the paddle in the power phase. In the phase of pulling the paddle out of the water the boat velocity is already being reduced and during the air work phase the boat velocity reaches its lowest values (Caplan, 2008; Hunter et al., 2008). This process is caused by friction forces (canoe – water) and the effect of air resistance on the canoeist and canoe above the water as well as by the fact that at that moment the participant only carries the paddle in the air and does not drive the boat by paddling. The participant uses whole body movement in his technique implementation. At the end of the air work phase he confers the boat an impulse, which helps to increase boat velocity. This trend of acceleration occurs in all monitored paces. The results show that intra-individual technique of the monitored participant changes, according to the selected paddling pace, only in a minimum way, mostly in the temporal frequency of individual strokes. At the starting paces, paddling frequency is higher and the boat velocity increases. In contrast, at distance paces we may notice the trend of consistent pace and even the canoe velocity remains unchanged. Only at the distance of 1000 m, decrease of velocity is apparent, which was probably caused by rising fatigue because recording was obtained at 800 m after the start. Fatigue factor was not examined directly in terms of internal load (heart rate, lactate, etc.). The evaluation of time spatial values (athlete – boat system), fatigue effect (various types of paddling pace) was not proved in our study. In further research, it would be appropriate to focus on simultaneous monitoring of internal load indicators.

Trajectories of individual segments in the XY and XZ plane are nearly identical at all distance paces. Only in the XY direction there are apparent differences at the starting pace. During the individual rides, the differences in the positions of segments between the strokes and their deviations are only minimal.

## Conclusion

The movement pattern was not significantly different at any paddling pace. There is the trend of almost smooth and straight drives. Even though the stroke is performed only on one side, almost direct movement of the canoe is maintained due to control manoeuvring strokes. Movement of the tip of the boat at the start is lower in the vertical direction than at other paces. This is caused by a faster frequency of individual strokes. The participant performs strokes in rapid succession and does not move the body as much as at other types of paces. In terms of time sequence there is a visible difference mainly at the pace after the start. We may conclude that inhibitory elements of the movement of the boat in the XY plane are resistance on the contact surface of the canoe with water, air resistance, athlete’s performance after pulling the paddle out of the water and the start of the air work phase related to the transfer of the paddle. On the other hand, movements of the torso and the upper extremities with the paddle in the final stage are the source of movement in the forward direction, which precedes the moving mechanism during the power phase. Paddling technique of the observed canoeist has been at a stable level thanks to many years of training. This indicates that the competitor is in the fourth phase of motor learning, where the specific physical activity has already been automated and therefore the external manifestation of the movement structure does not change.

## Figures and Tables

**Table 1:** Maximum and minimum velocities of selected points (m.s^−1^). Values are expressed as mean (standard deviation)

	**Start**	**Flying start**	**200 m**	**500 m**	**1000 m**
**Left hand**					
max. velocity	7.4 (0.1)	8.5 (0.2)	9.1 (0.2)	8.6 (0.4)	8.4 (0.4)
min. velocity	0.6 (0.1)	1.5 (0.1)	2.0 (0.1)	1.9 (0.1)	1.6 (0.1)
**Tip of the boat**					
max. velocity	4.5 (0.1)	5.2 (0.2)	6.1 (0.2)	5.9 (0.2)	5.4 (0.1)
min. velocity	2.1 (0.1)	3.2 (0.1)	3.9 (0.1)	3.9 (0.1)	3.0 (0.1)

**Table 2:** Vertical displacement of selected points (m). Values are expressed as mean (standard deviation)

	**Start**	**Flying start**	**200 m**	**500 m**	**1000 m**
**Right hand**	0.77 (0.02)	0.73 (0.02)	0.87 (0.03)	0.89 (0.03)	0.81 (0.04)
**Left hand**	0.57 (0.02)	0.55 (0.01)	0.69 (0.03)	0.63 (0.02)	0.70 (0.03)
**Head**	0.26 (0.01)	0.25 (0.01)	0.30 (0.02)	0.33 (0.02)	0.32 (0.02)
**Tip of the boat**	0.13 (0.01)	0.10 (0.01)	0.17 (0.01)	0.14 (0.01)	0.10 – 0.22 (0.01 –0.02)
